# Upregulation of LncRNA BCYRN1 promotes tumor progression and enhances EpCAM expression in gastric carcinoma

**DOI:** 10.18632/oncotarget.23585

**Published:** 2017-12-21

**Authors:** Hao Ren, Xiaomin Yang, Yongmei Yang, Xiaoyu Zhang, Rui Zhao, Ran Wei, Xin Zhang, Yi Zhang

**Affiliations:** ^1^ Department of Clinical Laboratory, Qilu Hospital, Shandong University, Jinan, Shandong Province, China; ^2^ Department of Laboratory, Yuhuangding Hospital, Qingdao University Medical College, Yantai, Shandong Province, China; ^3^ Department of Clinical Laboratory, The Affiliated Hospital of Qingdao University, Qingdao, Shandong Province, China; ^4^ Clinical Medicine of Undergraduate, Taishan Medical University, Taian, Shandong Province, China; ^5^ Wakayama Medical University, Wakayama, Wakayama, Japan

**Keywords:** BCYRN1, lncRNA, gastric carcinoma, tumor progression, EpCAM

## Abstract

Brain cytoplasmic RNA 1 (BCYRN1), along non-coding RNA, plays a critical role in various diseases, including some cancers. However, the expression of BCYRN1 and its roles in gastric carcinoma (GC) still remain unidentified. Thus, this study employed RT-qPCR to detect expression of BCYRN1 in 85 paired GC samples and adjacent normal tissues, and performed *in vitro* studies to explore effects of BCYRN1 in GC cells on cell proliferation, apoptosis and migration. We found BCYRN1 was significantly upregulated in GC samples, and its expression was positively correlated with advanced TNM stage (*p* = 0.0012) and tumor size (*p* = 0.027). Functionally, BCYRN1 knockdown by siRNA could inhibit cell proliferation, induce G1/G0 cell cycle arrest, increase apoptosis and impair migratory ability of AGS cells. Moreover, the results of RT-qPCR and western blotting indicated that knockdown of BCYRN1 notably decreased the expression of epithelial cell adhesion molecules (EpCAM). Otherwise, overexpression of BCYRN1 in GC cells (BGC-823 and SGC-7901) could reverse the effects of BCYRN1 knockdown. Taken together, our data indicate for the first time that BCYRN1 acts as an oncogenic lncRNA in GC progression and may be a potential therapeutic target in GC.

## INTRODUCTION

Gastric carcinoma (GC) is the fourth most common cancer and the second leading cause of cancer-related death worldwide [1]. Most GC patients are diagnosed at the advanced stages accompanied with malignant phenotype, characterized by excessive proliferation, relentless invasion local and distant metastasis [2]. Usually, these patients could not get effective treatment and have poor prognosis. Better understanding of the molecular mechanism underlying gastric carcinogenesis and progression will help to improve diagnosis and treatment of GC. Deregulation of tumor-related genes has been proved to play a significant role in the oncogenic processes, and these genes may serve as novel biomarkers and potential therapeutic targets for gastric cancer [2, 3].

Long non-coding RNAs (lncRNAs) are a group of transcripts longer than 200 nucleotides, having little or no protein coding capacity [4, 5]. It has been reported that lncRNAs contribute substantially to biological functions, such as regulating DNA transcription, modulating gene activity and remodeling chromosome structure [6–8]. Recently, lncRNAs have gained importance due to their deregulated expression in many diseases [9–11]. Brain cytoplasmic RNA 1 (BCYRN1) is a 200 nucleotide lncRNA, specifically transcribed in brain, neurons and germ cells [12, 13]. Compared with normal brains, brains of 70%patients with Alzheimer disease have decreased BCYRN1 expression, suggesting an important role ofBCYRN1 in neurodegeneration [14]. Because the neuronal specificity of BCYRN1 expression, it is rarely expressed in non-neural organs [15]. However, recent studies have found up-regulated BCYRN1 expression in several carcinoma tissues when compared with the corresponding adjacent normal tissues [16–18]. It has also reported that BCYRN1 is critically involved in the proliferation, apoptosis and metastasis of cancer cells [16, 17, 19, 20]. However, the expression of BCYRN1 and its biological functions in GC have rarely been investigated.

In current study, expression of BCYRN1 in GC tissues was detected and its correlations with GC patients’ clinical pathological characteristics were also analyzed. Then, we investigated the effects of BCYRN1 knockdown and overexpression in GC cells on cell proliferation, apoptosis and migration. In addition, correlation between BCYRN1 and epithelial cell adhesion molecule (EpCAM) was also assessed, since EpCAM is transcribed by BCYRN1's neighboring protein coding gene and is closely related to carcinogenesis and progression in most tumors [21–24]. Based on the analysis of our experimental data, this study presents the first evidence that BCYRN1 is upregulated in GC and acts as an oncogene to promote tumor progression and increase EpCAM expression.

## RESULTS

### BCYRN1 was highly expressed in both GC tissues and cell lines

**Figure 1:**
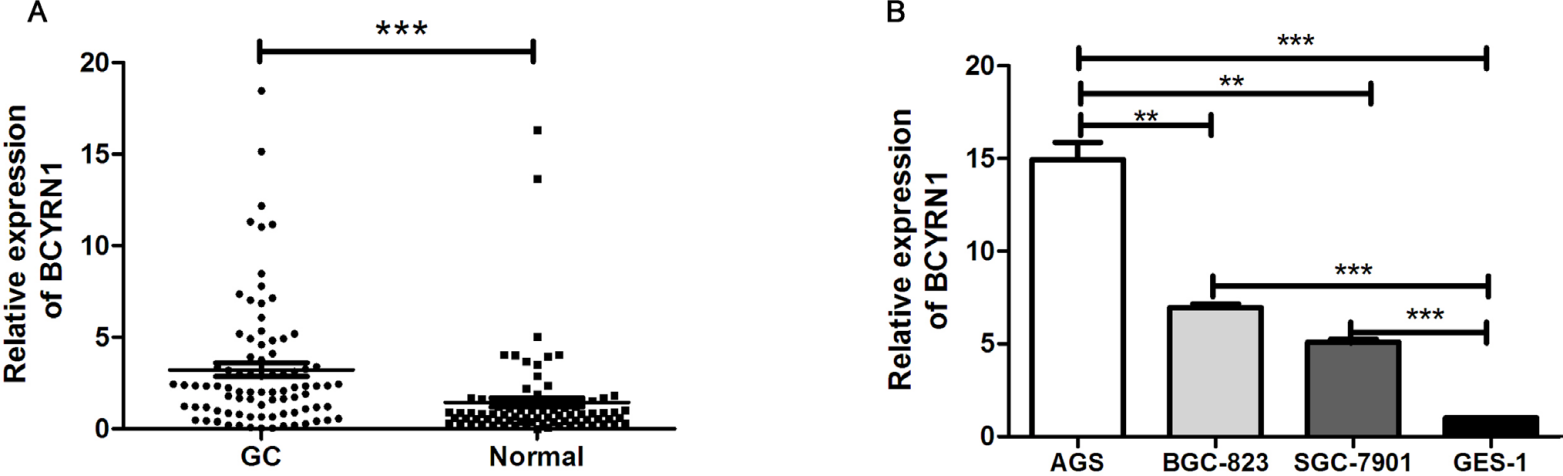
BCYRN1 expression in primary GC tissues and cell lines (**A**) The expression levels of BCYRN1 were examined by RT-qPCR in human GC tissues and their paired adjacent normal gastric tissues. Data represented the median (interquartile range) from three individual experiments. (**B**) The expression levels of BCYRN1 were detected by RT-qPCR in 3 GC cell lines and a normal gastric epithelial cell line. Data represented the median (interquartile range) or the mean ± SD from three individual experiments. ^**^p < 0.01, ^***^p < 0. 001.

We firstly detected BCYRN1 expression in 85 paired GC samples and adjacent normal tissues by RT-qPCR. The results showed that BCYRN1 expression was significantly higher in GC tissues than that in adjacent normal tissues (*p* < 0. 001, Figure 1A). Further analysis revealed that BCYRN1 expression was significantly associated with TNM stage (*p* = 0.0012) and tumor size (*p* = 0.027), while no significant relationship was observed between BCYRN1 and other clinic-pathological features, such as sex, age and distant metastasis (Table 1). We also found that three GC cell lines (AGS, BGC-823, SGC-7901) similarly displayed a high expression of BCYRN1 when compared to normal gastric epithelial cell line (GES-1) (all at *p* < 0.001, Figure 1B).

**Table 1:** Association of BCYRN1 and clinic-pathological features of GC patients

Variables	*n*	lncRNA BCYRN1 expression^a^	*p*-value
Gender			0.429
Male	56	2.076 (0.828, 4.115)	
Female	29	2.621 (1.214, 5.158)	
Age(years)			0.216
≥60	37	2.008 (0.703, 3.799)	
<60	48	2.347 (1.089, 6.857)	
TNM stage			0.0012^**^
I-II	27	1.070 (0.443, 2.534)	
III-IV	58	2.365 (1.605, 5.137)	
Lymph node metastasis			0.186
N0	16	1.559 (0.558, 3.082)	
N1 or above	69	2.343 (1.199, 4.927)	
Distant metastasis			0.125
M0	75	2.076 (0.859, 3.933)	
M1	10	4.096 (1.723, 6.600)	
Tumor differentiation			0.373
Poor	50	1.783 (0.810, 4.521)	
Moderate	26	2.364 (1.311, 4.594)	
Well	9	2.972 (2.029, 4.826)	
Tumor size			0.027^*^
<5	39	1.626 (0.568, 3.472)	
≥5	45	2.347 (1.430, 6.468)	

^a^Data represent the median with interquartile range.^*^p < 0.05, ^**^p < 0.01.

### BCYRN1 promoted cell proliferation and cell cycle progression in GC cell lines

**Figure 2:**
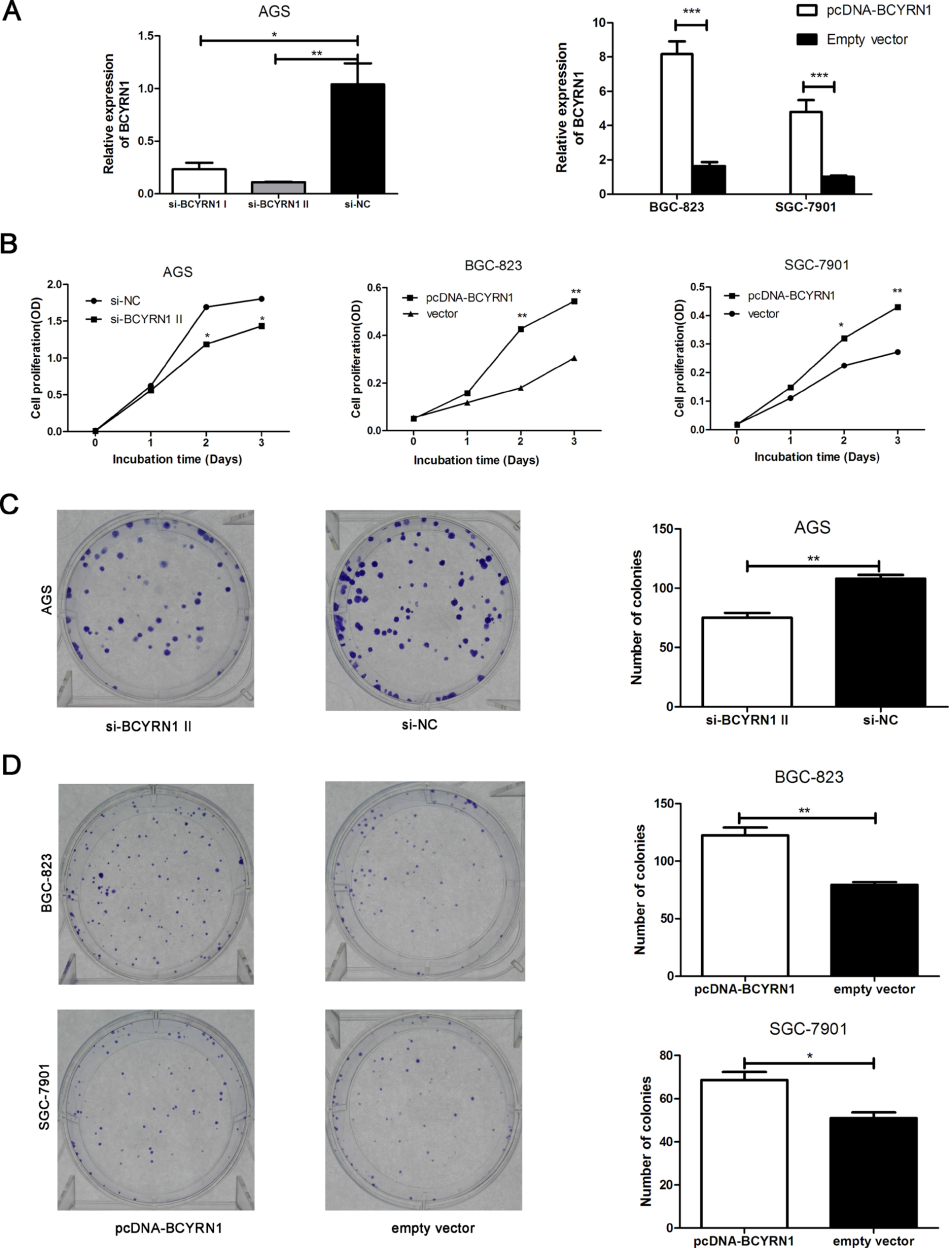
Effect of BCYRN1 on GC cell proliferation AGS cells were transfected with siRNAs against BCYRN1 (si-BCYRN1I and si-BCYRN1 II) or negative control siRNA (si-NC) for 24 h. BGC-823 and SGC-7901 cells were transfected with plasmid vector of BCYRN1 (pcDNA-BCYRN1) or empty vector for 24 h. (**A**) Knockdown and overexpression of BCYRN1 were confirmed by RT-qPCR in these three GC cell lines, respectively. On the basis of the RT-qPCR result, si-BCYRN1 II was employed for all experiments unless otherwise indicated. (**B**) CCK-8 assay was used to detect the cell proliferation abilities of these three GC cell lines, respectively. (**C**, **D**) The ability of colony formation was detected in transfected cells (AGS, BGC-823 and SGC-7901), respectively. Data represented the mean ± SD from three independent experiments.^*^p < 0.05, ^**^p < 0.01, ^***^p < 0.001.

**Figure 3:**
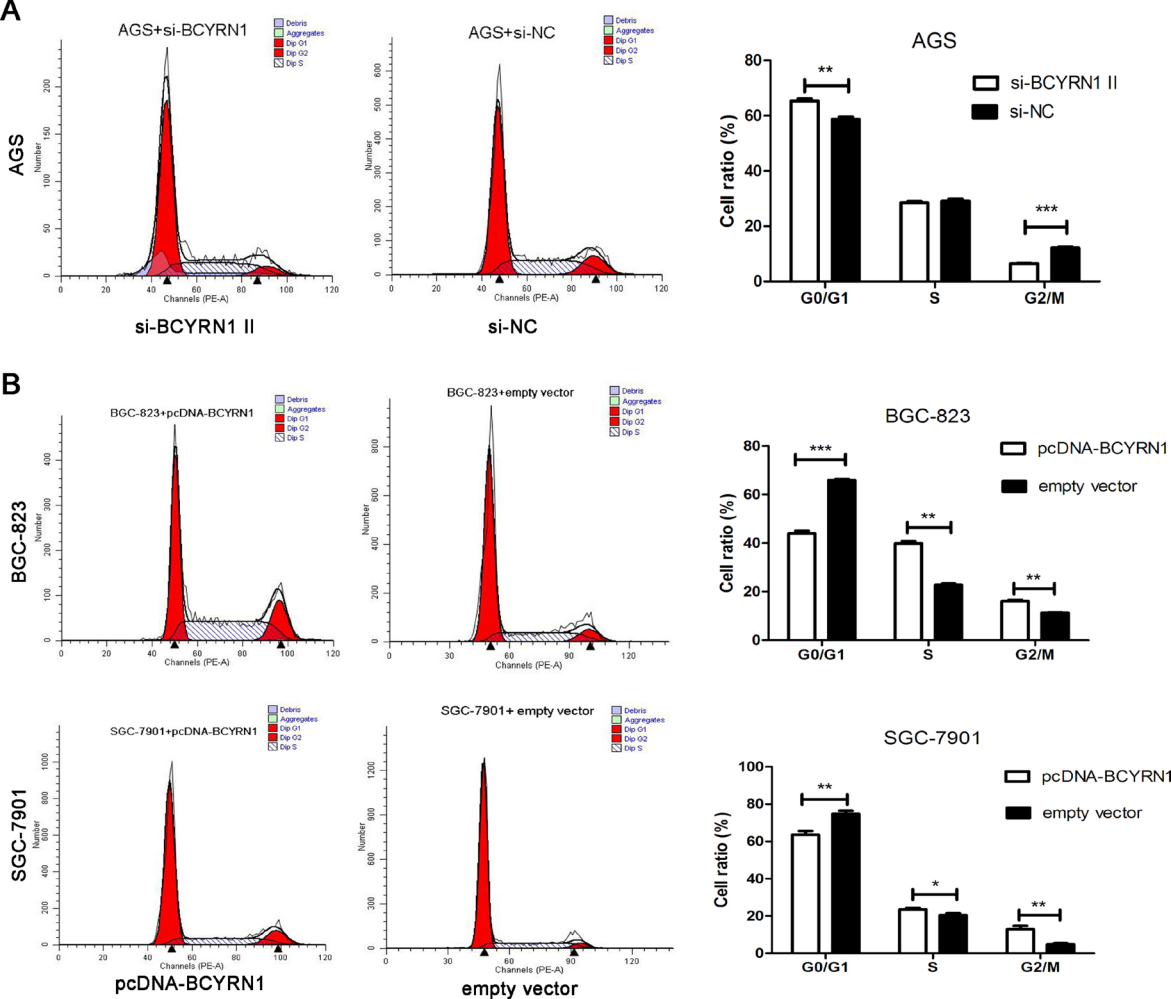
Effect of BCYRN1 on GC cell cycle At 48 h post transfection, flow cytometry was used to determine the effect of knockdown or overexpression of BCYRN1 on cell cycle of GC cell lines. (**A**) Cell cycle of AGS cells transfected with si-BCYRN1 and si-NC. (**B**) Cell cycle of BGC-823 and SGC-7901 cells transfected with pcDNA-BCYRN1 and empty vector. Representative data were expressed as the mean ± SD from three independent experiments.^*^p < 0.05, ^**^p < 0.01, ^***^p < 0.001.

We performed a loss-of-function experiment using si-BCYRN1 in AGS cells with higher BCYRN1 expression, and did the reverse using pcDNA-BCYRN1 to BGC-823 and SGC-7901 cells with lower BCYRN1 expression (Figure 1B). Efficiencies of knockdown and overexpression were confirmed by RT-qPCR (Figure 2A). CCK-8 assays showed that knockdown of BCYRN1 inhibited cell proliferation, while forced its expression promoted cell proliferation (Figure 2B). Colony-formation assays revealed that knockdown ofBCYRN1 significantly decreased the colony forming ability of AGS cells (Figure 2C). In addition, overexpression of BCYRN1 could boost the number of BGC-823 and SGC-7901 cell clones, respectively (Figure 2D). Furthermore, flow cytometry analysis showed that knockdown of BCYRN1 increased the G0/G1 phase population in AGS cells (Figure 3A), while overexpression of BCYRN1 increased the G2/M phase population and reduced the G0/G1 phase population in BGC-823 and SGC-7901 cells (Figure 3B).

### BCYRN1 suppressed apoptosis of GC cells under serum-deprived condition

**Figure 4:**
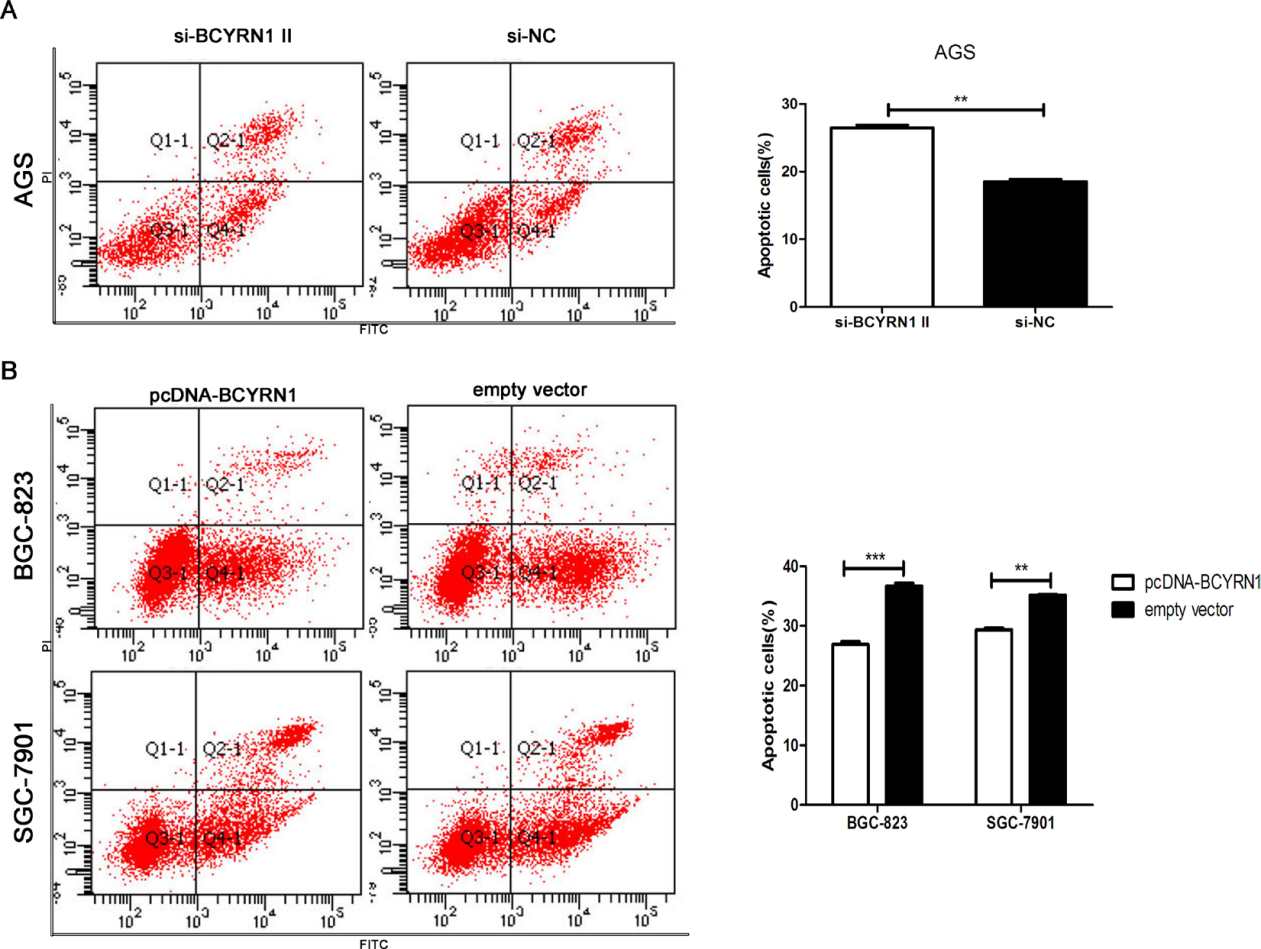
Effect of BCYRN1 on GC cell apoptosis under serum-deprived condition After overnight serum starvation, flow cytometry was used to detect the apoptosis of transfected GC cells, and early apoptotic cells were shown in gate Q4-1. (**A**) Apoptosis of AGS cells transfected with si-BCYRN1 and si-NC. (**B**) Apoptosis of BGC-823 and SGC-7901 cells transfected with pcDNA-BCYRN1 and empty vector. Three individual experiments were done, and representative data were expressed as the mean ± SD. ^**^p < 0.01, ^***^p < 0.001.

To explore whether BCYRN1 has a role in regulating apoptosis of GC cells, we performed flow cytometric analysis of serum starvation-induced apoptosis following BCYRN1 knockdown or overexpression in GC cells. After overnight serum starvation, knockdown of BCYRN1 increased the apoptotic population of AGS cells (Figure 4A). Meanwhile, overexpression of BCYRN1 inhibited apoptosis of BGC-823 and SGC-7901 cells (Figure 4B).

### BCYRN1 promoted GC cell migration

**Figure 5:**
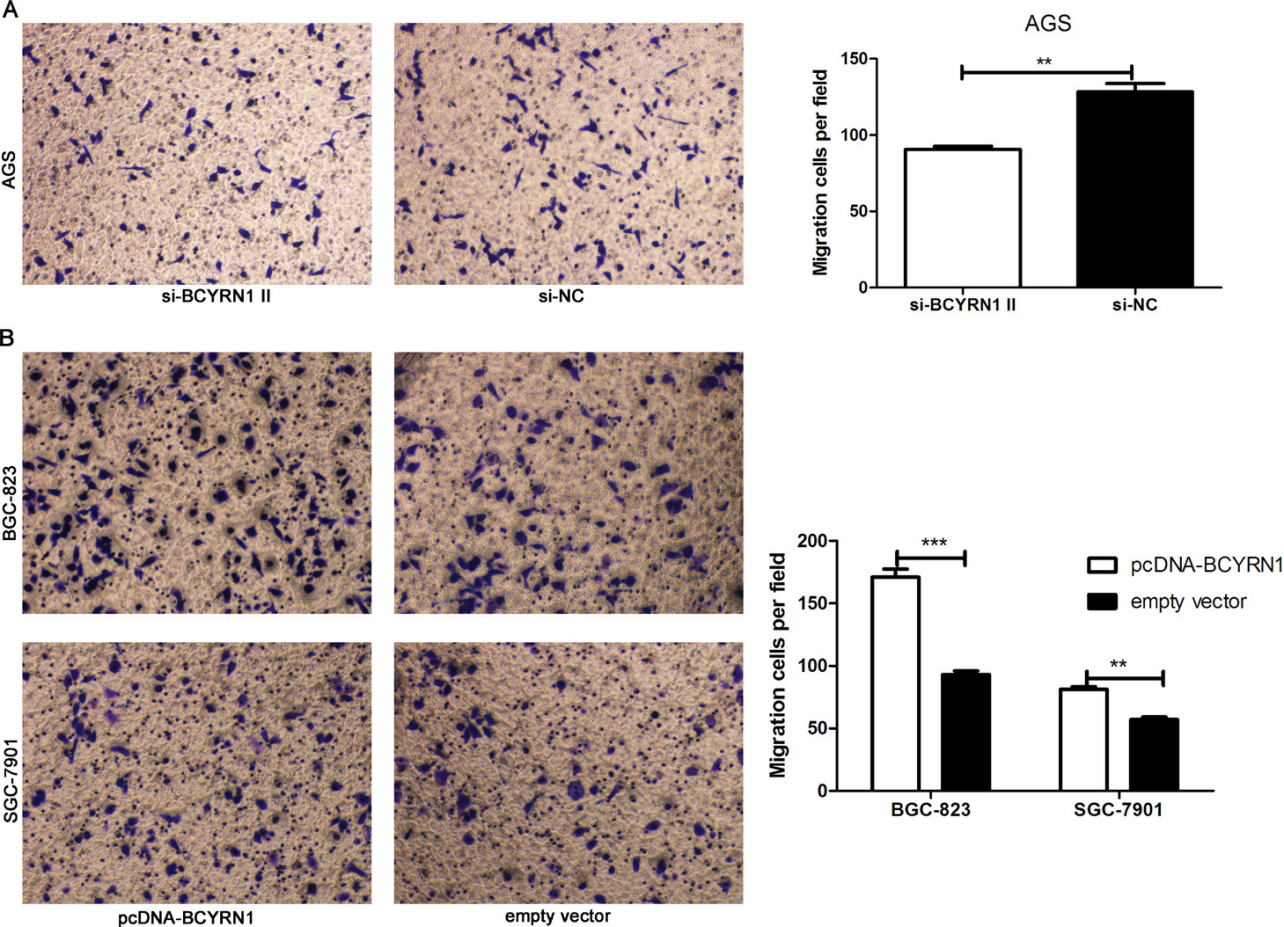
Effect of BCYRN1 on cell migration Transwell migration assay was used to evaluate the migratory abilities of transfected GC cells. (**A**) The migration abilities of AGS cells transfected with si-BCYRN1 and si-NC. (**B**) The migration abilities of BGC-823 and SGC-7901 cells transfected with pcDNA-BCYRN1 and empty vector, Three individual experiments were performed, and representative data were expressed as the mean ± SD.^**^ *p* < 0.01,^***^p < 0.001.

BCYRN1 was reported to regulate the cell motility in different types of cells [17, 20]. Here, we employed transwell assay to assess the effect of BCYRN1 on GC cell migration. Our results showed that knockdown of BCYRN1 could significantly inhibit the migration activities of AGS cells (Figure 5A), while overexpression of BCYRN1could significantly stimulate the migration activities of BGC-823 and SGC-7901 cells (Figure 5B).

### BCYRN1 upregulated the expression of EpCAM in GC cells

**Figure 6:**
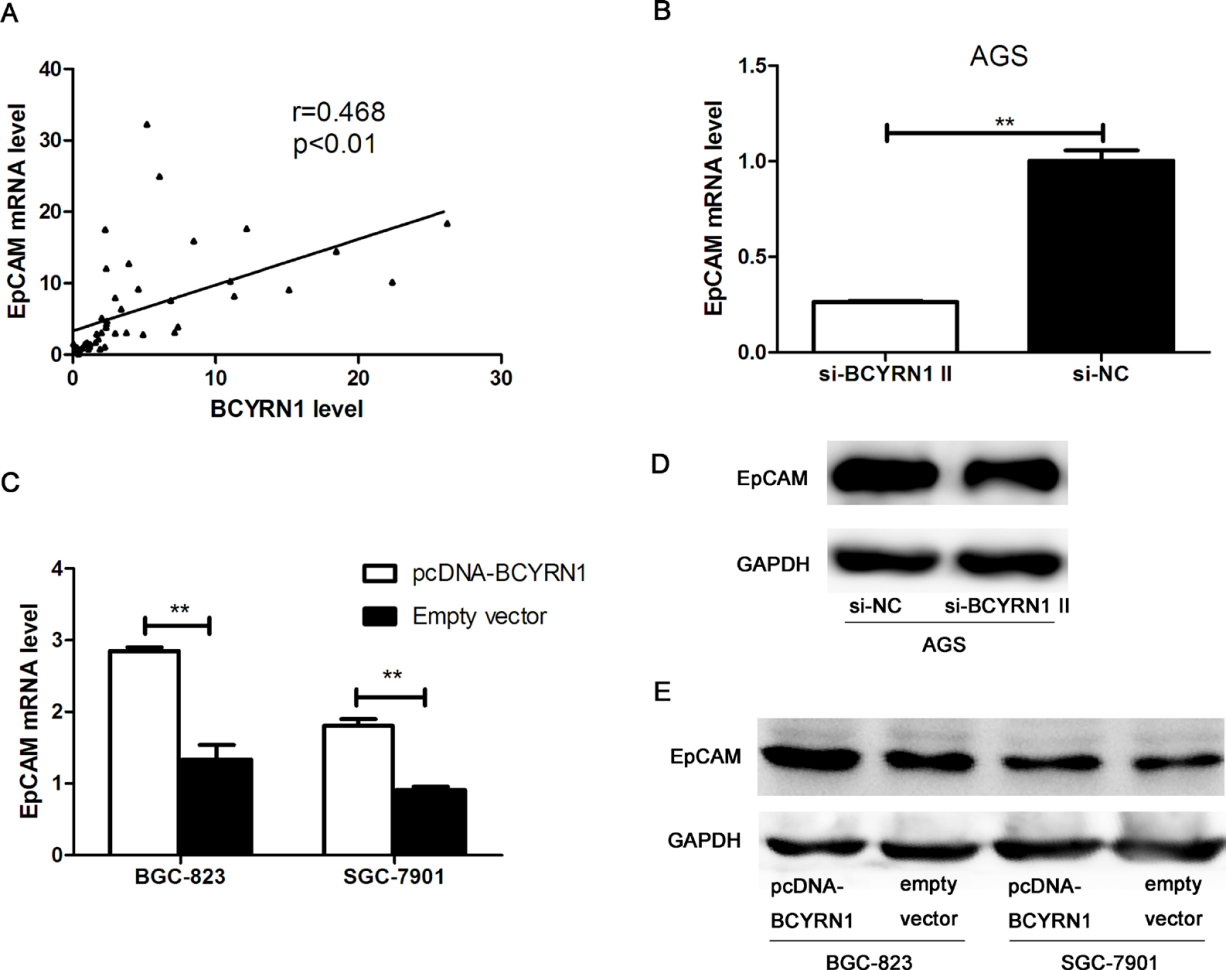
The role of BCYRN1 in regulating EpCAM expression (**A**) The positive linear correlation between BCYRN1 transcription and EpCAM mRNA expression in GC tissues. (**B**) The level of EpCAM mRNA in AGS cells transfected with si-BCYRN1 and si-NC. (**C**) The level of EpCAM mRNA in BGC-823 and SGC-7901 cells transfected with pcDNA-BCYRN1 and empty vector. (**D**) Representative western blot images of EpCAM detection in AGS cells transfected with si-BCYRN1 and si-NC. (**E**) Representative western blot images of EpCAM detection in BGC-823 and SGC-7901 cells transfected with pcDNA-BCYRN1 and empty vector. Data presented as representative images or the mean ± SD of three independent experiments. ^**^p < 0.01, ^***^p < 0.001.

We retrieved information from the National Center for Biotechnology Information (NCBI) Gene database (http://www.ncbi.nlm.nih.gov/gene) and found that the EpCAM gene is located near BCYRN1. EpCAM is highly expressed in most cancers with an epithelial origin and acts as an oncogenic signaling protein [16, 17, 20]. We hypothesized that BCYRN1 might upregulate EpCAM expression and thus further stimulate oncogenicity of GC. Clinical data showed their expressions have a positive correlation in 85 cases of GC tissues (Figure 6A). Using RT-qPCR and western blot, we then confirmed that the expression of EpCAM was significantly enhanced in BCYRN1-overexpressed BGC-823 and SGC-7901 cells (Figure 6B, 6D), while knockdown of BCYRN1 resulted in the down-regulation of EpCAM in AGS at both the mRNA and protein levels (Figure 6C, 6E).

## DISCUSSION

In recent years, dozens of lncRNAs have been shown to play critical regulatory roles in cancer biology [25]. In the present study, we showed that the increased expression of BCYRN1 promoted cell proliferation and migration, and suppressed apoptosis in gastric carcinoma. In addition, we demonstrated that BCYRN1 might regulate the expression of EpCAM, which is closely related to carcinogenesis and progression of cancers [22, 26]. For the first time, we found that BCYRN1 could act as an oncogenic lncRNA in the development of GC.

BCYRN1 is a cytoplasmic lncRNA transcribed by RNA polymerase III [13]. There are some unique features of this RNA: (i) its expression can be found only in primates; (ii) it is specifically transcribed in brain, neurons and germ cells, and transported to postsynaptic dendrites; (iii) it works as a regulator of translation initiation, by interacting with poly(A)-binding protein [12, 13, 27]. Recently, high levels of BCYRN1 in breast cancer tissues, esophageal squamous cell carcinoma tissues and non-small-cell lung cancer tissues have been observed [16–18]. In the present study, we provided the first evidence that BCYRN1 was highly expressed in GC and significantly correlated with high TNM stages and large tumor size. Our data indicated the possible roles of BCYRN1 in pathogenesis and progression of GC. These findings suggested that tumor cells might misappropriate the unique mechanisms of translational control in neurons and germ cells and thus the deregulation of BCYRN1 arose.

Deregulated proliferation is one of the hallmarks of tumor cells and also a cause of their rapidly development [3]. Mistakes in cell cycle process possibly lead to unrestrained cell proliferation and may ultimately facilitate malignant transformation [28]. Our results showed that there were remarkable shifts of cell proliferation after the expression of BCYRN1 in GC cells was changed. We demonstrated for the first time that up-regulated BCYRN1 resulted in a reproducible accumulation of cells in S and G2/M phases, combined with observably decreased percentages in G1/G0 phase. We have postulated that GC cells might drive tumor growth through a mechanism involving BCYRN1-mediated cell proliferation. Moreover, we found BCYRN1 could also evade serum starvation induced apoptosis, consistent with its role of regulating apoptosis in breast cancer cells [19]. Herein, our data indicated that BCYRN1 might contribute to gastric carcinogenesis by accelerating cell proliferation and inhibiting cell apoptosis.

BCYRN1 have been reported to regulate cell motility in different types of cells. For example, BCYRN1 could upregulate the expression of transient receptor potential and thus promoted migration of rat airway smooth muscle cells in asthma [29]. In HeLa cervical carcinoma cells, depletion of BCYRN1 disrupted the cellular motility by destabilizing mRNA for calcium-binding protein S100A11 [30]. In non-small-cell lung cancer cells, the number of migrated and invaded cells and the expression of metastasis-supporting proteins MMP9 and MMP13 changed with the regulation of BCYRN1 [17]. Consistent with these studies, our results showed that knockdown and overexpression of BCYRN1 in human GC cell lines reduced and stimulated the migration activities of GC cells, respectively. These findings suggested an important role for BCYRN1 in cancer metastasis [16, 17].

Several studies have discovered that some lncRNAs can regulate their neighboring protein coding genes’ expression by cis-effection [6, 10, 31]. BCYRN1 is just a promoter upstream transcript of its neighboring protein coding gene EpCAM [21]. EpCAM has been shown to exert critical functions in cell proliferation, differentiation, apoptosis and migration of diverse cell types [22, 32–34] and it also plays an important role in GC development [35–38]. In this study, we hypothesized that BCYRN1 might regulate EpCAM and could thus exert similar effects on GC cells. Our data demonstrated that BCYRN1 up-regulated expression of EpCAM at both the mRNA and protein level. And this process was accompanied by functional alterations in GC cells, indicating BCYRN1 might exert its biological effects on GC cells, at least partially, by the regulation of EpCAM.

Although substantial expression and biological functions of BCYRN1 in GC had been illustrated in our study, there were still a number of limitations. First, it was unclear whether BCYRN1 play its role through regulating EpCAM. Second, with our present data, we couldn't confirm whether there are other genes involved in BCYRN1-mediated signal pathway. Thus, elucidating the subtle molecular mechanism of BCYRN1 to promote GC progression may require more powerful evidences in future work.

In conclusion, we demonstrate that BCYRN1 acts as an oncogenic lncRNA to promote cell proliferation, migration and suppress apoptosis, and simultaneously enhance the expression of EpCAM in GC. Our study provides new insights regarding cancer progression and highlights the potential utility of BCYRN1 as a novel therapeutic target in GC.

## MATERIALS AND METHODS

### Patients and clinical samples

A total of 85 paired GC tissue and adjacent normal gastric tissue specimens were collected from patients who had underwent radical resection at Qilu Hospital of Shandong University between March 2015 and October 2017. All specimens were immediately frozen and stored in liquid nitrogen until RNA extraction. The clinical features and pathologic classifications of all patients were recorded in Table 1. Tumors were staged according to the 2010 Union for International Cancer Control (UICC) TNM staging system. We had got the approval of the Ethics Committee of Qilu Hospital, and the study was carried out after all patients involved in this research had signed written informed consents.

### Cell culture

Three human GC cell lines, including AGS, BGC -823, SGC-7901, and a human normal cell line GES-1(human gastric epithelium-immortalized cell) were obtained from the Culture Collection of the Chinese Academy of Sciences (Shanghai, China). BGC-823, SGC-7901 and GES-1 cells were grown in RPMI-1640 medium (Hyclone, USA) containing 10% fetal bovine serum (FBS) (Hyclone, USA), while AGS cells were cultured in F12 medium (Hyclone, USA) containing 10% FBS in a 5% CO_2_ humidified incubator at 37°C.

### Total RNA extraction and cDNA Synthesis

Total RNA was extracted from frozen tissues or cell lines using TRIzol reagent (Invitrogen, USA) in accordance with the manufacturer's protocol. The concentration of total RNA was determined by ultraviolet-visible spectrophotometer, and absorbance was measured at 260 nm. The cDNA was synthesized from 500 ng total RNA using Prime Script™ RT reagent Kit (Takara, China).

### RT-qPCR

The quantitative real-time polymerase chain reaction (RT-qPCR) was performed using the SYBR Premix Ex Taq™ II (Takara, China) in the CFX-96 real-time PCR System (BIO-RAD, USA). The thermal cycling program of this reaction was as follows: an initial denaturation step (95°C for 15 sec), 40 cycles (95°C for 5 sec, 60°C for 30 sec) and melting curve analysis. The BCYRN1, EpCAM and GAPDH primers were as follows: BCYRN1 forward, c-CTCAGGGAGGCTAAGAGGCG-3′; BCYRN1 reverse, 5′-TTTCCTTTTTCTGGAGAACGGG-3′; EpCAM forward, 5′-TCAGAAGAACAGACAAGGAC-3′; EpCAM reverse, 5′-ACTGCTATCACCACAACCAC-3′; GAPDH forward, 5′-AGCCACATCGCTCAGACAC-3′; GAPDH reverse, 5′-GCCCAATACGACCAAATCC-3′.

The melting curve was used to determine the specificity of the RT-qPCR product. The specificity of RT-qPCR products was evaluated with melting curve analysis and the relative expression of genes was calculated by the 2^-ΔΔCt^ method. The reference gene GAPDH was used as an endogenous control to normalize the results in our experiments.

### Transfection

Cells were seeded in 24-well plates at 5×10^4^ per well. After 24 hours serum free incubation, plasmid vector of BCYRN1 (pcDNA-BCYRN1) (Gene Chem, China) and empty vector were transfected into BGC-823 and SGC-7901 cells with Lipofectamine 2000 reagent (Invitrogen, USA) according to the manufacturer's instructions. BCYRN1-specific siRNA (si-BCYRN1) and negative control siRNA (si-NC) (Gene pharma, China) were transfected into AGS cells in the same way. The sequences of si-BCYRN1 were as follows: si-BCYRN1 I, 5′-CGUUCUCCAGAAAAAGGAATT-3′; si-BCYRN1 II, 5′-GUAACUUCCCUCAAAGCAATT-3′.

### Cell proliferation assay

Cell proliferation was quantified using the Cell Counting Kit-8 (CCK-8) (Beyotime, China). Twenty four hours after transfection, cells were plated in 96-well plate (4 × 10^3^ cells/well) and incubated overnight. Two hours after the medium of each well was replaced by freshly prepared medium with CCK-8, the absorbance of wells was measured on 0, 24, 48 and 72 hour with ELISA reader (Thermo Scientific, USA) at 490 nm.

### Colony formation assay

Transfected cells were seeded in six-well plate (300 cells/well) and maintained in medium with 10% FBS for 2 weeks. Colonies were fixed with methanol for 15 minutes and stained by crystal violet. The number of individual colonies was counted using microscope.

### Flow cytometry for cell cycle

Forty eight hours after transfection, cells were washed three times with the cold phosphate-buffered saline (PBS) and fixed by 75% ethanol at −20°C for 1 hour. Then the fixed cells were weighted in 200 μl cold PBS and stained with presidium iodide (BD Bioscience, USA) 500 μl for 15 minutes in the dark. The cell cycle was analyzed by FACSCalibur instrument (BD Bioscience, USA) and software named ModFit.

### Serum starvation apoptosis analysis

To assess apoptosis, 24 hours after transfection, cells were rinsed 3 times with PBS and incubated in medium without serum overnight. Then 400 μl blocking solution, 5 μl annexin V–FITC and 10 μl PI were added into collected cells, the mixture was incubated for 15minutes without light. The cell apoptosis was detected by flow cytometry (BD Bioscience, USA).

### Cell migration assay

Forty eight hours after transfection, 10 × 10^4^ cells suspended in 200 μl serum-free medium, were plated in the upper chamber of 24-well transwell chambers (pore size: 8mm) (BD, USA). Seven hundred μl medium with 20% FBS was added to the lower chamber as an attractant. After 28 hours of incubation, cells were stained with crystal violet for counted.

### Western blot

Seventy two hours after transfection, cells were lysed with RIPA lysis buffer (Beyotime, Beijing, China) for 30 min on ice. Protein was electrophoresed on 10%SDS-PAGE and blotted to PVDF membranes. Membranes were blocked in TBST buffer with 5% fat-free milk and incubated separately with primary anti-EpCAM monoclonal antibody (1:1000, Cell Signaling Technology, USA) or anti-GAPDH monoclonal antibody (1:1000, Abcam, USA) at 4°C. After overnight incubation and washing, membranes were incubated with secondary antibody (1:5000, Zhongshan Goldenbridge, China) at room temperature for 2 h. Western Chemiluminescent HRP Substrate (Millipore, USA) was used to visualizing the immunoblots.

### Statistical analysis

Kolmogorov-Smirnov test was used to determine the distribution of each group. Skew distributed data were expressed as median and interquartile range, and normally distributed data were described as mean ± SD. Differences between two groups were performed with Mann-Whitney *U*-test or Student's *t*-test, as appropriate. Kruskal-Wallis *H* test was conducted for comparison among three groups. The association between BCYRN1 and EpCAM mRNA was examined by Spearman correlation analysis. Statistical analyses were processed with SPSS 19.0 software (IBM, USA), and figures were performed using GraphPad Prism 5 software (La Jolla, USA). All experiments were separately preformed in triplicate. Results were considered to be statistically significant at *p* < 0.05.
